# Metabolic Acidosis Results in Sexually Dimorphic Response in the Heart Tissue

**DOI:** 10.3390/metabo13040549

**Published:** 2023-04-12

**Authors:** Yamin Liu, Amina Atiq, Anna Peterson, Mikayla Moody, Ashkan Novin, Alix C. Deymier, Junaid Afzal, Kshitiz

**Affiliations:** 1 Department of Biomedical Engineering, University of Connecticut Health, Farmington, CT 06032, USA; yamin.liu@uconn.edu (Y.L.);; 2 Division of Cardiology, Department of Medicine, University of California San Francisco, San Francisco, CA 94158, USA

**Keywords:** metabolic acidosis, dilated cardiomyopathy, cardiac contractility, chronic cardiac damage, sexual dimorphism, acidosis, low-grade acidosis, sex differences, heart transcriptomics, acidosis model

## Abstract

Metabolic acidosis (MA) is a highly prevalent disorder in a significant proportion of the population, resulting from imbalance in blood pH homeostasis. The heart, being an organ with very low regenerative capacity and high metabolic activity, is vulnerable to chronic, although low-grade, MA. To systematically characterize the effect of low-grade MA on the heart, we treated male and female mice with NH_4_Cl supplementation for 2 weeks and analyzed their blood chemistry and transcriptomic signature of the heart tissue. The reduction of pH and plasma bicarbonate levels without an associated change in anion gap indicated a physiological manifestation of low-grade MA with minimal respiratory compensation. On transcriptomic analysis, we observed changes in cardiac-specific genes with significant gender-based differences due to MA. We found many genes contributing to dilated cardiomyopathy to be altered in males, more than in females, while cardiac contractility and Na/K/ATPase-Src signaling were affected in the opposite way. Our model presents a systems-level understanding of how the cardiovascular tissue is affected by MA. As low-grade MA is a common ailment with many dietary and pharmaceutical interventions, our work presents avenues to limit chronic cardiac damage and disease manifestation, as well as highlighting the sex differences in MA-induced cardiovascular damage.

## 1. Introduction

Metabolic acidosis (MA) is a common disorder resulting from imbalance of pH homeostasis in the blood, affecting millions of people worldwide [1]. Human blood is slightly basic, with a narrow physiological range of pH 7.35–7.45, owing to tight regulation by body’s multiple buffer systems [1]. This non-neutral basic pH is essential to facilitating oxygenation of the blood and other metabolic processes in the body. Amongst the three main buffers of the blood, the bicarbonate buffer system, which is coupled with respiration, is the most important component for regulation of physiological pH homeostasis, maintaining a slightly positive bicarbonate/carbonic acid ratio [2]. MA is primarily characterized by a reduction in bicarbonate concentration, along with a decrease in blood pH, which can last from days (acute) to weeks or longer (chronic) [1,3]. The reduction in pH results from one of several disorders caused by either increased acid production in body, reduced excretion of acid through kidneys, and/or loss of bicarbonate [1,3]. The main clinical causes of MA are diabetes, cancer, liver failure, alcoholism, a high protein diet, chronic kidney disease, and aging. MA is also caused by loss of bicarbonate in hyperchloremic acidosis due to gastrointestinal, renal, or other causes such as dietary electrolyte imbalance and responses to pharmacological compounds [1,4,5,6,7].

Disruption of the key buffering systems results in high-grade acidosis; however, pH may remain balanced at values closer to the lower limit of the normal range (7.35) at the cost of bicarbonate stores depletion, which is categorized as low-grade acidosis [8,9]. Chronic, low-grade MA is largely ascribed to high acid-producing modern diets, excessive consumption of acid precursor foods, lack of base precursors, and aging [10,11,12]. At low severity, chronic MA mostly remains undetected, but likely affects a large proportion of the population. Although the rapid buffering capacity within the body results in correction of changes in pH, even a slight long-term decrease in pH has been shown to have a significant impact on various bodily parameters, including loss of muscle mass and bone mineral content [13,14,15,16]. Chronic MA, even at low-grade, also contributes to clinical outcomes such as increased risk of hypertension and type 2 diabetes [17,18]. Chronic MA, even at low-grade, is known to enhance catabolic pathways and proteolysis in skeletal muscle through aberrant neural and hormonal factors, an adaptive mechanism necessary for the supply of amine groups/glutamine for the renal compensation of acidosis [16,19,20,21]. Although, several studies have established the effect of chronic low-grade MA on skeletal muscle wasting [13,14,15,16,19,20], its effect on cardiac muscle is not well known. Contractility of the heart is sensitive to even slight changes in pH [22]. Acid–base disturbance can severely impair heart function, even without prior cardiovascular disease, and predispose the heart towards arrhythmia [23]. A large retrospective study on the effects of non-dialysis chronic kidney disorder, including more than 50,000 patients, showed that lower serum HCO_3_^−^ was associated with a higher risk of MACE+ (major adverse cardiovascular events) incidence [24]. However, the mechanisms by which MA causes cardiovascular impairment are unclear.

To understand how systemic MA can affect the heart tissue, we sought to characterize the transcriptomic effect of reduced HCO_3_^−^ in a mouse model of chronic low-grade acidosis. Our mouse model of chronic acidosis involves stepwise increase in NH_4_Cl administration via the drinking water, which is humane, unlike nephrectomies [25], as well as allowing for examination of the effect of both acute and chronic MA. We have previously reported that, upon diet administration of this diet, mice recreate the essential features of clinical acidosis, including decrease in blood pH, aberrant bone remodeling and increased skeletal fragility, as well as loss of bone carbonate [26]. To study the effect of acidosis on the heart tissue, we used our model with induced acidosis and studied the gene expression in cardiac tissue. Further, there is increasing appreciation of the differences in cardiac disease manifestation between male and female subjects [27]. We therefore separately looked at differences in male and female heart gene expression after 2 weeks of acidosis in our model.

Surprisingly, we found that male and female heart tissue responded differently to systemic low-grade MA, an effect that is also observed in human skeletal muscle wasting in response to chronic low-grade MA [28]. Both at the level of individual gene expression, as well as key pathways related to cardiac function, there were differences between the male and female response to systemic acidosis. Specifically, we found that male and female hearts showed opposite effects on genes associated with cardiac contractility. Female hearts were more responsive to estrogen response signaling after acidosis. Our report will help to identify the molecular mechanisms involved in sex differences in cardiac gene expression and highlight the importance of accounting for sexual dimorphism in managing MA.

## 2. Materials and Methods

### 2.1. Animals

The study protocol was approved by the Animal Care and Use Committee at the University of Connecticut Health Center, Farmington, CT and all experiments were performed according to National Institutes of Health Guide for the Care and Use of Laboratory Animals (NIH Publications No. 8023, revised 1978). Healthy male and female CD1 mice (aged 5–6 months) were purchased from Charles River Laboratories (Wilmington, MA, USA). Mice were kept in a 12 h light–dark cycle, temperature-controlled (22 ± 2 °C) and humidity-controlled (55 ± 5%) environment and fed a standard chow diet for at least 2 weeks before the start of experimentation.

### 2.2. Induction of Metabolic Acidosis

Male and female CD1 mice were evenly distributed into two experimental groups, control and acidosis. Each group contained 4 mice. The metabolic acidosis mouse model was induced as described previously [26]. Briefly, on the first day of acidosis induction, day 0 (D0), the drinking water for the acidosis group was replaced with an aqueous solution of 0.2 M ammonium chloride (NH_4_Cl) and 5% sucrose. The NH_4_Cl dose in the drinking water was then increased by 0.1 M every 3 days for up to 14 days. The mice were allowed to consume solid food and liquid diet ad libitum. Control mice remained on standard drinking water for the duration of the experiment.

### 2.3. Blood and Urine Chemistry and Assessment of Acidosis

Blood samples were collected at baseline (D-7) and day 14 (D14) to measure pH and ion levels. Briefly, 200–300 μL of blood was extracted from non-anesthetized mice through submandibular puncture procedures. Blood samples were analyzed using a Heska POC Epoch Blood Gas Analyzer (Love-land, CO, USA) to obtain values of blood pH, partial pressure of O_2_ (pO_2_), partial pressure of CO_2_ (pCO_2_), blood urea nitrogen (BUN), total carbon dioxide (cTCO_2_), base excess of extracellular fluid (BE), base excess of blood (BE(b)), oxygen saturation (csO_2_), and ion concentrations of actual bicarbonate (HCO3^−^), calcium, sodium, and chloride, anion gap blood (AGap), and metabolic indices including lactate (Lac), hematocrit (Hct), hemoglobin (cHgb), creatinine, and glucose. Urine samples were collected via manual expression from mice at D0, D3, D6, D9, D12, and D14, from which the pH was determined using Hydrion pH strips with a resolution of 0.1 pH units. On day 14, the mice were killed and their hearts were harvested and rapidly frozen in liquid nitrogen for RNA sequencing.

### 2.4. RNA Sequencing and Analysis

Total RNA was extracted from the flash frozen mice heart tissue using a RNeasy Mini Kit (Qiagen, Hilden, Germany) according to manufacturer’s protocol. RNA integrity was estimated using Bioanalyzer 2100 (Agilent, Santa Clara, CA, USA) and samples with RIN~8 were sent to Novogene Inc. (Sacramento, CA, USA). for library preparation and RNA sequencing. Raw FASTQ data were aligned to the mouse genome index (GRCm39) using the HISAT2 pipeline with default parameters [29,30]. Reads were counted using HTSeq, and fold changes and statistical significance (*p*-values) for differentially expressed genes were calculated using DESeq2 [29,30]. For each functional category, gene sets were used from the Gene Ontology (GO), KEGG, Msigdb and WikiPathways to select the genes in the transcriptomic analysis [29,30].

Hierarchical clustering with Euclidean distance (UPGMA method) was performed on z-scores calculated on TPM (transcripts per million) normalized data [29,30]. Ontologies of genes in each cluster, separated on hierarchical clustering, were evaluated using GO, WikiPathways, KEGG and Reactome using gprofiler2 [29,30]. Differentially expressed genes were used to calculate up/downregulation of canonical pathways and transcription factors (TransFac) using Ingenuity Pathway Analysis (IPA) from Qiagen Inc (Qiagen, Hilden, Germany). Gene Set Enrichment Analysis (GSEA) [31] was performed on differentially expressed genes, using WebGestalt [32] and fgsea [33], using methods published earlier [29,30].

### 2.5. Statistical Analysis

Blood gas, urine PH and body weight are expressed as mean ± SD unless otherwise noted. Two-tailed Student’s *t*-tests were used to compare data from acidosis mice to their respective controls. Mixed model ANOVA with a Geisser–Greenhouse correction was performed to evaluate the differences between groups at a specific timepoint. Statistical significance is defined as *p* < 0.05 (*), *p* < 0.01 (**) or *p* < 0.001 (***).

## 3. Results

### 3.1. Stepwise NH_4_Cl Dietary Supplementation Recreates Clinical Features of Metabolic Acidosis

**Figure 1:**
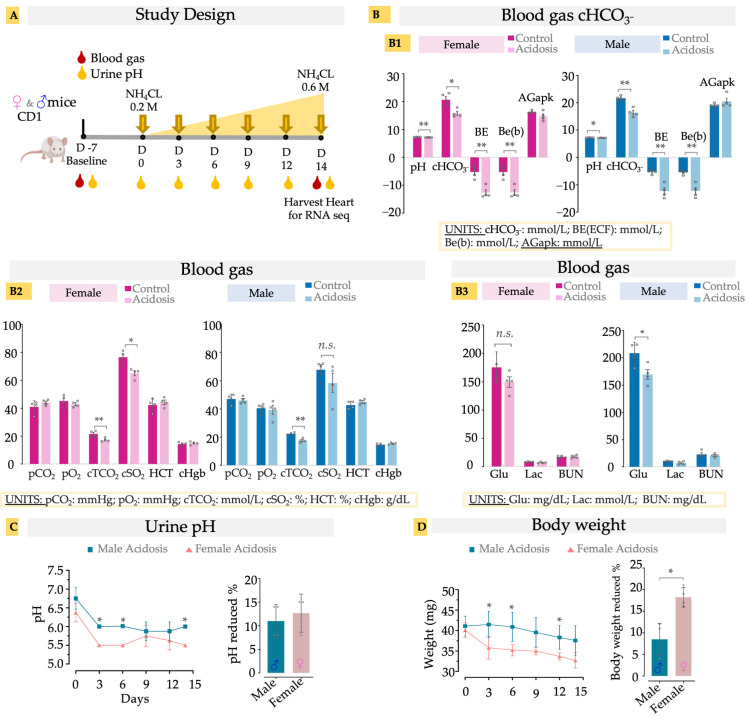
(**A**) Schematic of study design. Oral administration of NH_4_Cl, using stepwise dosing increase, induces murine acidosis. Urine pH was measured at baseline (D-7), D0, D3, D6, D9, D12, and D14. Blood gas was measured at baseline (D-7) and D14 for both control (n = 4) and acidosis (n = 4) groups. (**B**) Blood gas measurement. **B1**, pH, HCO_3_^−^, BE, Be(b) and AGapK levels in the blood; **B2**, pCO_2_, pO_2_, cTCO_2_, cSO_2,_ cHgb, Hct level in blood; **B3**, Glu, lac and BUN level in blood. (**C**) urine pH, (**D**) body weight. Two tailed student’s *t*-test was used to determine differences between acidosis and control. * < 0.05; ** < 0.01, n.s. (non-significant). Actual bicarbonate (cHCO_3_^−^; mmol/L), base excess of extracellular fluid (BE(ecf); mmol/L), base excess of blood (BE(b); mmol/L), Anion gap (AGapK, mmol/L), Carbon dioxide partial pressure (pCO_2_; mmHg), oxygen partial pressure (pO_2_; mmHg), total carbon dioxide (cTCO_2_; mmol/L), oxygen saturation (cSO_2_; %), hematocrit (Hct; %PCV), hemoglobin (cHgb; %), Glucose (Glu; mg/dL), lactate (Lac; mmol/L), Blood urea nitrogen (BUN; mmol/L).

As previously reported [26,34], the administration of NH_4_Cl successfully induced acidosis by reducing blood pH and HCO_3_^−^ levels in the blood of the dosed mice compared to controls (Figure 1(A,B1)). The reduction in excess base, pH and plasma bicarbonate levels without affecting the anion gap (AGapK) indicates a low-grade metabolic acidosis (MA), in both male and female mice model, caused by the exogenous NH_4_Cl (Figure 1(B1)). Acidosis is known to cause a rightward shift in the oxygen dissociation curve (ODC), an adaptive response described by the Bohr effect, which results in reduced oxygen saturation at the given partial pressure of oxygen to facilitate higher oxygen availability to the tissues [35]. We observed that our model of chronic low-grade MA also exhibited reduced oxygen saturation (cSO_2_) without affecting the partial pressure of O_2_ (pO_2_) (Figure 1(B2)). The effect was statistically significant in female mice, while most male mice also showed a similar reduction (Figure 1(B2)). Total CO_2_ concentration (cTCO_2_), which is the sum of all forms of carbon dioxide in the blood, was also reduced in both male and female acidosis mice (Figure 1(B2)). As the partial pressure of the CO_2_ (pCO_2_) was not affected, the reduced ctCO_2_ reflects reduction of plasma bicarbonate levels, indicating MA without respiratory compensation. Hematopoietic factors such as hematocrit and hemoglobin were unaffected (Figure 1(B2)), as has been observed earlier in rodent models of MA [36]. Similarly, lactate and BUN were also not changed with MA, suggesting a minimal effect of MA on kidney function, while male acidosis mice showed a negligible reduction in glucose levels (Figure 1(B3)).

Urine pH exhibited a significant decrease in pH with administration of NH_4_Cl (Figure 1C). This reduction was more significant in the females than males at D3, D6, and D14, but the overall drop in pH was not different based on sex (Figure 1C). Administration of NH_4_Cl caused a significant decrease in body weight (8–18%), with the females losing significantly more weight than the males over the same time course (Figure 1D). Together, these results suggest that the NH_4_Cl dietary model successfully replicates clinical low-grade metabolic acidosis, while avoiding secondary health effects.

### 3.2. Differential Gene Expression of the Heart Reveals Dimorphic Acidosis-Induced Changes in Male and Female Heart Tissue

**Figure 2:**
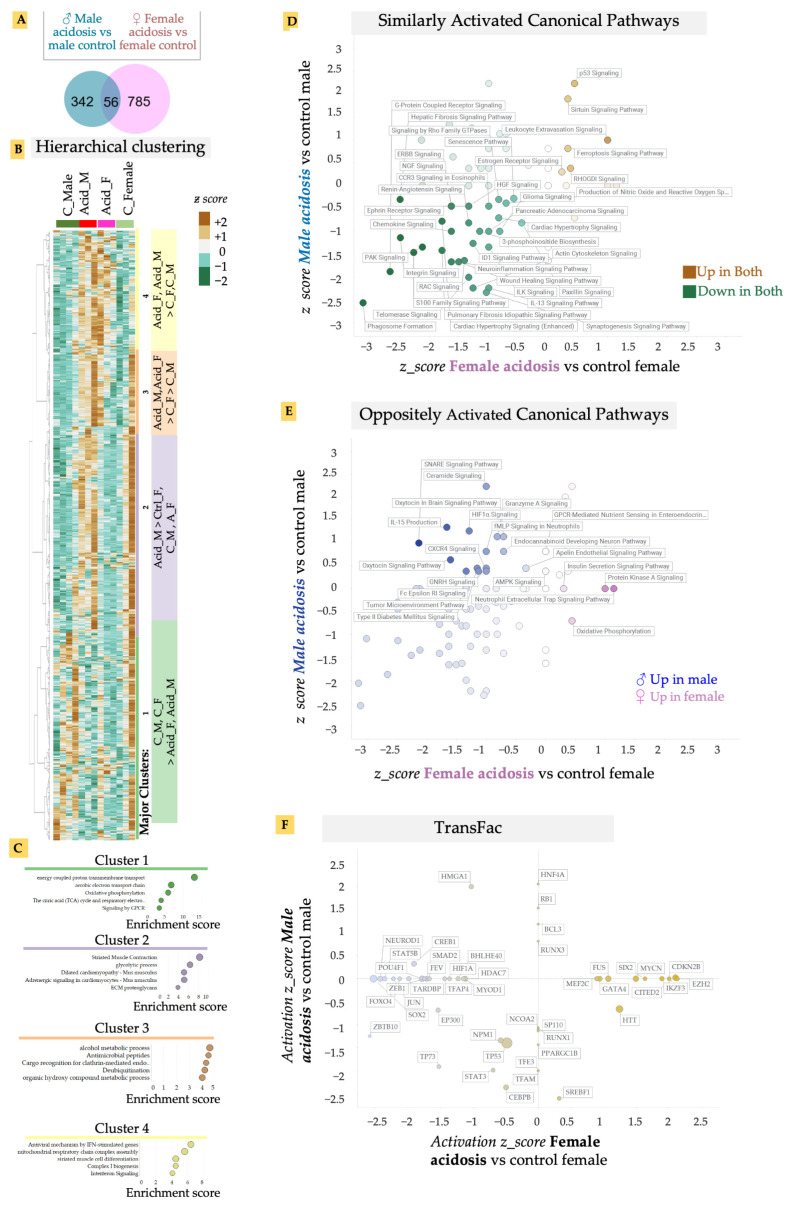
(**A**) Venn diagram of differentially-expressed genes in male and female acidosis heart vs. their respective controls. (**B**) Unbiased hierarchical clustering using z-scores of all expressed genes show four main clusters. Acidosis results in upregulation of genes in Cluster 3 and 4, while Cluster 1 is upregulated in control mice hearts. (**C**) Ontology analysis on genes in the four main clusters. (**D**) Quadrant map of canonical pathways that are up/downregulated in both male and female acidosis model. (**E**) Canonical pathways that are oppositely regulated in male vs. female acidosis mice model. (**F**) Quadrant map of transcriptional factors (TransFac) activated/inhibited in male vs. female acidosis mice model. The canonical pathways and transcription factors were calculated using IPA analysis of differentially expressed genes.

We isolated fresh heart tissue from both male and female mice after 2 weeks of induced acidosis and isolated RNA. mRNA was sequenced and gene expression analyzed. Low-grade MA resulted in a relatively larger change in differential gene expression in the female mice compared to males, when matched with their respective controls (Figure 2A). There was also considerable variability among individual cohorts, suggesting that lowered blood pH was either variably buffered in the heart or the effect of pH was variably regulated (Figure 2A). Surprisingly, we found only a small overlap in the MA vs. control gene expression between male and female cohorts, suggesting a sexually dimorphic response to low-grade MA on the heart (Figure 2A). Hierarchical clustering of genes revealed four major distinct clusters in differential gene expression patterns, in an unbiased analysis of expressed genes (Figure 2B). Identifying gene ontologies enriched in each major cluster, we found that genes that reduced in expression in MA, in both male and female hearts (Cluster 1), were associated with metabolic and energetic pathways, as well as G-protein coupled receptor signaling (GPCR) (Figure 2C). High contractility of cardiomyocytes necessitates a high energy consumption, requiring highly efficient metabolism [37]. The theory that MA resulted in decreased activation of energetic pathways in both male and female hearts is borne out by previous reports that ATP production and cardiac contractility, as well as energy substrate utilization, is decreased in the heart by acidosis [38,39]. G-protein coupled receptors (GPCR) and their signaling plays an essential role in cardiovascular biology [40,41]. Adrenergic, muscarinic, angiotensin, and endothelin receptors are amongst several of the GPCRs in the heart that are known to regulate cardiac contractility/rate, growth, and enlargement of myocytes, and are dysregulated in almost all cardiac diseases [40,41]. Cluster 4 refers to genes that were upregulated in response to MA in both males and females, and contained inflammation-related gene ontologies, particularly those activated by interferon signaling, as well as biogenesis of electron transport chain subunits.

To analyze the effect of MA on cardiac gene expression in more detail, we calculated the activated pathways in MA separately in male and female hearts using Ingenuity Pathway Analysis (IPA). IPA confirmed that GPCR signaling-related pathways were downregulated in both male and female hearts in response to acidosis (Figure 2D). Indeed, while acidosis resulted in activation of a few pathways, key cardiovascular-related signaling was downregulated in both male and female hearts. In particular, we noted that MA resulted in significant and large inactivation of several pathways related to vascular regulation (Figure 2D). These included reduced activation of renin angiotensin signaling, a critical long term regulator of systemic blood resistance and hypertension [42], p21 activated kinases (PAK) signaling involved in vascular integrity and arrhythmias [43], and IL-13 signaling, which is essential in repair and structural homeostasis of the heart [44,45,46].

MA also resulted in significant reduction in pathways related to cardiac contractility in both male and female hearts. These included downregulation of enhanced cardiac hypertrophy signaling, RAC signaling, which contributes to cardiomyocytes’ polarity and contractility [47], integrin and paxillin signaling, which are important for electromechanical coupling in the heart [48], and signaling by the Ca^2+^ binding S100 family of proteins [49]. We have previously shown that presentation of the matrix ligands specific to integrin receptors expressed in the adult heart can facilitate rapid maturation of differentiated cardiomyocytes [29]. Surprisingly, we also found downregulation of pro-fibrotic signaling in the heart reflected in downregulation of the wound healing pathway, as well as the idiopathic fibrosis pathway. Although a few pathways were similarly activated by MA in both male and female mice, sirtuin signaling stood out. Sirtuins are NAD+ dependent histone deacytelases that are important for metabolism in many tissues, including in the heart. Sirtuin signaling can provide cardioprotection to redox stress, as well as vascular damage [50]. We also found increased p53 activation, which is correlated with increased apoptosis due to cardiac failure [51], or dysregulation of the proteasomal system due to dilated cardiomyopathy [52], suggesting that MA may have induced a pathological response in the heart, leading to increased apoptosis.

To specifically analyze the sexually dimorphic response of acidosis in the heart, we separated out IPA pathways that showed an oppositely directional activation in male and female hearts after MA (Figure 2E). While the pro-survival IL-15 pathway that signals to natural killer cells was only upregulated in males, it was downregulated by MA in females [53]. Males also displayed higher inflammation due to MA, with increases in ceramide signaling [54], which induces damage to cardiomyocytes, leading to various cardiometabolic pathologies [54], as well as GNRH signaling, which is associated with cardiovascular events [55]. Interestingly, we also found increased anti-inflammatory and cardioprotective oxytocin signaling in males, while this was decreased in females [56].

Finally, we identified upstream regulators explaining the downstream gene expression changes with MA in both males and females (Figure 2F). Concentrating on the upstream transcription factors (TFs), we identified that many key cardiac TFs changed differently between male and female hearts.

### 3.3. Gene Set Enrichment Analysis of Male and Female Hearts in Response to Acidosis

**Figure 3:**
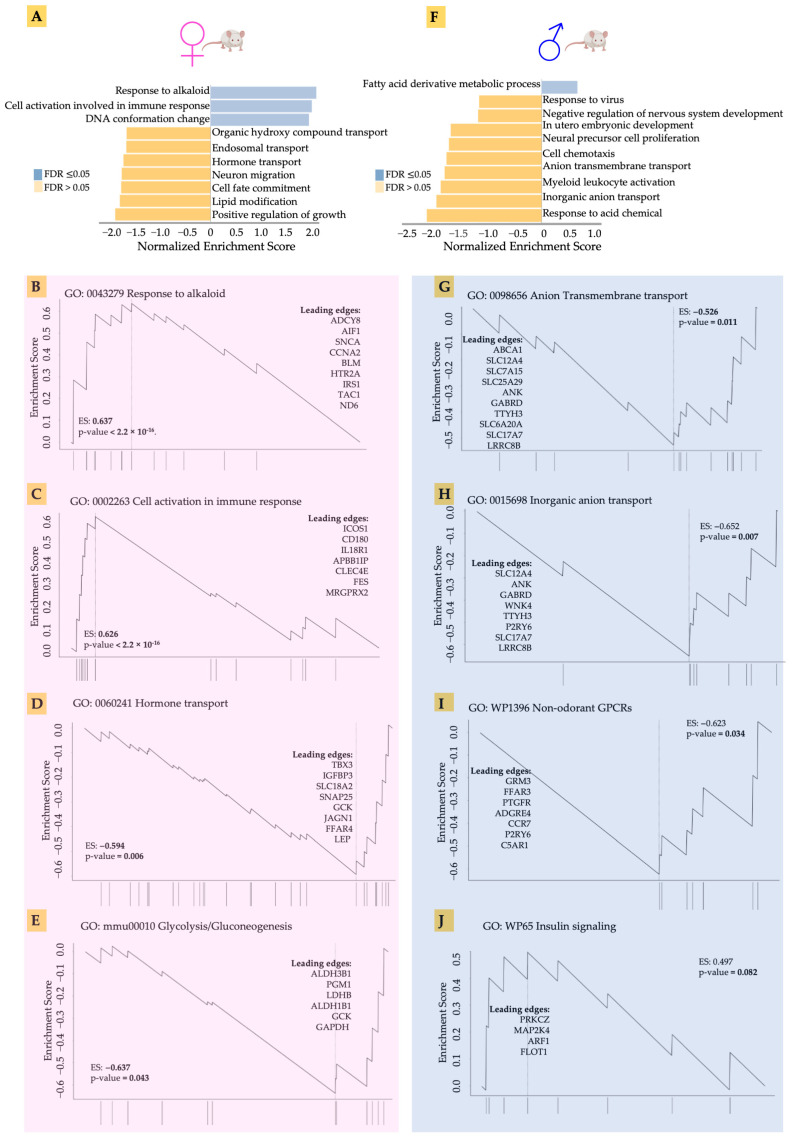
(**A**,**F**) The top enriched gene ontologies were very different between male and female samples, acccording to non-parametric Gene Set Enrichment Analysis (GSEA). (**B**–**E**) GSEA showing the leading edge genes in response to alkaloid, cell activation in immune response, hormone transport, and glycolysis in acidosis female mice compared to control female mice. (**G**–**J**) GSEA showing the leading edge genes in response to anion transmembrane transport, inorganic anion transport, wp1396 non-odorant GPCRs, and WP65 insulin signaling in acidosis male mice compared to control male mice.

Our preliminary analysis had indicated that acidosis affects gene expression in the heart tissue, as well as identifying differences between male and female hearts. We therefore set out to explore in greater detail the gene expression changes in males and females in response to acidosis. Using the non-parametric Gene Set Enrichment Analysis (GSEA), we found that the top enriched gene ontologies were very different between male and female samples (Figure 3). We found that acidosis resulted in increased enrichment of ontologies related to response to alkaloids, as well as cellular activation of immune response in females. The leading edge genes suggested activation of stress response in female hearts (Figure 3B). These genes included IRS1 encoding insulin receptor substrate, the major component of insulin signaling, the loss of which leads to heart failure [57], as well as ADCY8 encoding cAMP producing adenylate cyclase 8, which protects against cardiac stress [58]. We also found an increase in cell cycle-related genes among the leading edge, including CCNA2 and AIF1, likely suggesting increased vascular proliferation. Increased enrichment of cellular immune response in the female hearts was accompanied by increased expression of ICOS1, which is known to promote hypertension [59], a receptor to interleukin-18, which mediates cardiac dysfunction [60], and CLEC4E, levels of which correlate with myocardial injury in response to ischemia reperfusion [61].

Female hearts also showed a significantly large negative enrichment in hormone transport. The leading edge genes suggested significant reduction in cardiac regenerative functions (Figure 3D). These included TBX3, a transcription factor necessary for specification of the atrioventricular conduction system [62], and IGFBP3 encoding the IGF binding protein 3 responsible for heart regeneration [63]. We also found reduced glycolytic metabolism in female hearts in response to acidosis (Figure 3E). The leading edge genes included two isoforms of aldehyde dehydrogenase ALDH3B1 and ALDH1B1, as well as lactate dehydrogenase B (LDHB), which catalyzes the conversion of pyruvate to lactate.

Male hearts, in contrast, showed a very different pattern in gene ontology enrichment compared to the female hearts. Fatty acid oxidation was increased, while many gene sets were significantly negatively enriched. Anion and inorganic transport were negatively enriched (Figure 3G,H). Acidosis resulted in reduced expression of many genes encoding ion channels, including SLC12A4 encoding a potassium chloride cotransporter, sodium dependent phosphate carrier and many others. Interestingly, non-odorant GPCRs were negatively enriched in female hearts, with the leading edge constituting many genes related to the interaction of the sympathetic nervous system with the cardiac tissue (Figure 3I). These genes included GRM3 encoding glutamate metabotropic receptor 3 [64], and FFAR3, which encodes a receptor for short fatty acid 3 regulating neurohormonal control of circulation [65]. Insulin signaling was also decreased in the male hearts (Figure 3J), with key signaling leading edge genes constituting PRKCZ, which encodes protein kinase C2 and regulates phosphorylation of cardiac sarcomeric proteins [66], and the MAP2K4 encoding MAP kinase, kinase 4, which prevents a maladaptive response to hypertrophy [67]. Overall, GSEA analysis suggested that, while female hearts mounted a stress response to acidosis, male hearts showed decreased expression of key genes necessary for normal cardiac function.

### 3.4. Acidosis Results in a Sexually Dimorphic Effect on Cardiomyocyte Contractility, and Increased Transcription Related to Dilated Cardimyophathy in Males

**Figure 4:**
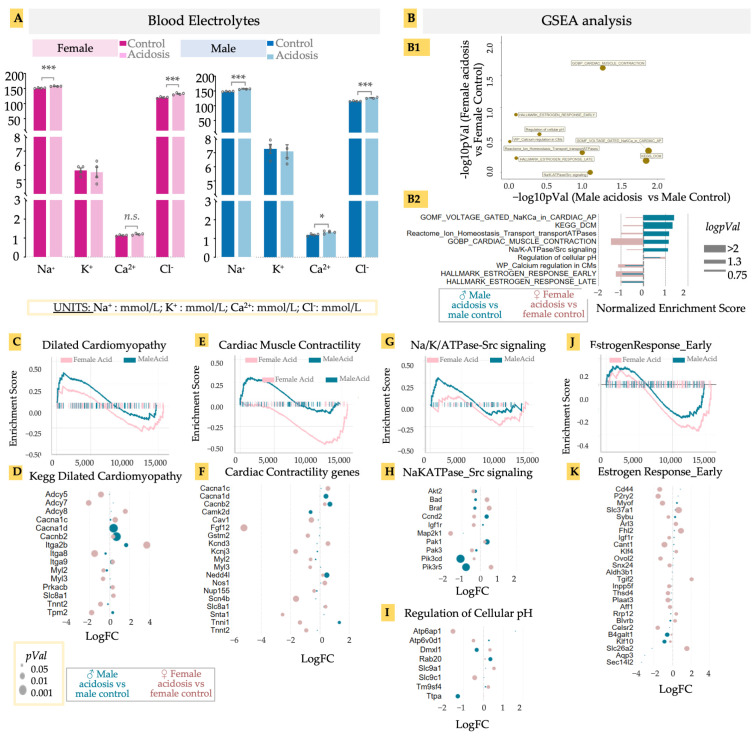
(**A**) Blood electrolytes. Acidosis compared to control; two-tailed student’s *t*-test was used to determine the significance. * < 0.05; *** < 0.001; n.s. (non-significant). Sodium (Na, mmol/L), potassium (K, mmol/L), calcium (Ca, mmol/L), chlorine (Cl, mmol/L). (**B**) GSEA analysis of differentially expressed genes in male and female acidosis models, (**B1**) Targeted GSEA analysis and negative logpVal of relevant/cardiac ontologies. (**B2**) enrichment scores of the relevant/cardiac ontologies in GSEA analysis. GSEA and bubble plot of differentially expressed genes in DCM ontology (**C**,**D**), Cardiac muscle contractility (**E**,**F**), and Na/K/ATPase-Src signaling (**G**,**H**). Bubble plot of differential genes in regulation of cellular pH (**I**). GSEA and bubble plot of differentially expressed genes in estrogen response early ontology (**J**,**K**). GSEA and bubble plots are color coded with male acidosis vs. male control (blue) and female acidosis vs. female control (pink). Size of bubble or bar plot is determined with either pVal (*p*-value), logpVal or−−log10 of pVal.

Low-grade acidosis resulting from only 2 weeks of NH_4_Cl supplementation resulted in significant changes in gene expression in the hearts, with many gene ontologies showing a sexually different activation score. Chronic acidosis has been known to cause changes in heart function, contributing to heart failure. Acidosis is expected to result in changed balance of ions in the blood, which could adversely affect cardiac function [68]. We therefore examined blood electrolytes in both males and females at the end of the experimental period, prior to killing the animals (Figure 4A). Chloride levels increased significantly in both males and females, likely as a result of the NH_4_Cl administration, while sodium levels showed different patterns of change. While Na+ levels decreased in females, they increased slightly in males with an even higher base. Changes in other electrolytes were not significant (Figure 4A). Electrolyte imbalances can lead to various cardiac pathologies, including the onset of arrhythmias [69,70]. However, could acidosis result in gene expression changes at the level of ion channels, probably as compensatory mechanisms? We looked at specific gene sets associated with ion channel-related cardiac functions in acidosis mice vs. control mice, separately for males and females, and calculated their *p*-values (Figure 4(B1)), as well as enrichment score (Figure 4(B2)). Several gene sets were upregulated in female hearts after acidosis but showed little change in male hearts, including those related to early and late estrogen response, calcium regulation, and ATPases associated with ion transport homeostasis. In contrast, the gene set associated with dilated cardiomyopathy was highly upregulated in males, as was voltage gated Na/K/Ca in action potential generation. Cardiac muscle contraction was activated differently in the sexes, with a strong activation in males and a strong inactivation in females (Figure 4B).

These differential analyses suggested that acidosis manifests in male and female hearts with different effects on gene expression related to cardiac diseases. Acidosis has been shown to adversely affect cardiac function, but a sexual dimorphism in acidosis-related cardiomyopathy has not previously been described. Concentrating on these identified cardiac disease- and functions-related gene sets, we identified the leading edge genes from GSEA analysis, and calculated the fold change in these genes in response to acidosis, separately in male and females. In the gene set “Dilated Cardiomyopathy” (DCM), male hearts had a moderate but significant enrichment (Figure 4C). Among the leading edge genes, we found increased expression in Tpm2 encoding beta-tropomyosin in males, mutations in which are associated with DCM phenotype [71] (Figure 4D). Tpm2 is also a predictive marker for atherosclerosis [72]. In contrast to males, female hearts showed a large reduction in Tpm2 expression in acidosis. We also found two key genes encoding voltage gated calcium channel, Cacna1d and Cacnb2, which were significantly increased in males, but not in females (Figure 4E,F). This is remarkable, because a Cacna1d inhibitor, verapamil, is under clinical trial for DCM patients (NCT00374465) [73,74]. To note, we also found a significant and large decrease in the adenylate cyclase encoding genes Adcy5 and Adcy7 in female hearts, which are known to contribute to protection from DCM [75,76], strongly suggesting that acidosis can change gene expression in the heart tissue, leading to the onset of dilated cardiomyopathy.

In the case of cardiac contractility, we found a clearly sexually dimorphic response. While female hearts were negatively enriched, male hearts were positively enriched (Figure 4E). This observation was borne out in the expression of key genes contributing to myocyte contractility. These included the voltage gated calcium channel unit mentioned earlier, Cacna1d, as well as a key cardiac ubiquitin ligase, Nedd4l, which regulates sodium channel activity, with its mutations involved in various cardiac diseases including DCM [77] (Figure 4F). We also found a large increase in expression of Tnni2 in males, encoding fast switch skeletal muscle Troponin I isoform. Interestingly, Tnni2 showed a large decrease in females. Of note, Fgf12, which is known to reduce heart remodeling [78], was reduced more than five-fold in females. Myosin light chain encoding genes Myl2 and Myl3 were also reduced in female hearts. Female hearts also showed increased expression in K+ channel subunit encoding Kcnd3, while inner K+ rectifier potassium channel Kcnj3 was reduced.

We also found significant enrichment in the gene set associated with Src signaling in response to Na/K/ATPase activity in male hearts in response to acidosis (Figure 4G), which has clinical significance in DCM [79]. Remarkable leading edge genes were Pik3cd and Pik3r5, encoding different catalytic subunits of phosphatidylinositol-4,5-bisphosphate 3-kinases, both of which were significantly reduced in males (Figure 4H). Another relevant gene set was regulation of cellular pH, showing acidosis-induced reduction of Atp6ap1 and Atp6v0d1 in females, both genes encoding for subunits of vacuolar H+-ATPase, and mutations that are associated with cardiac abnormalities [80] (Figure 4I).

Finally, we observed negative enrichment of early estrogen response in both male and female hearts in response to acidosis, with a greater effect size observed in females (Figure 4J,K). Few male-specific leading genes appeared with significant fold changes, with the notable exception being transcription factor encoding Klf10, whose reduction is associated with cardiac hypertrophy [81]. In female hearts, acidosis caused a large decrease in expression of Cd44, the hyaluronan receptor, which is critically involved in healing after infarct injury [82], as well as P2ry2, which encodes the purinergic-Y2 G-protein coupled receptor highly expressed in cardiac tissue, and is involved in key cardiovascular functions [83,84], not only in cardiac contractility but also fibroblast activation. Also notable was Tgif2, encoding TGF induced factor homeobox 2, which is involved in cardiac development [85]. Overall, these data provide key avenues to explore acidosis-induced change in cardiac transcriptomics and consequent changes in cardiac function, while highlighting a strong case of sexual dimorphism in cardiac disease manifestation.

## 4. Discussion

**Figure 5:**
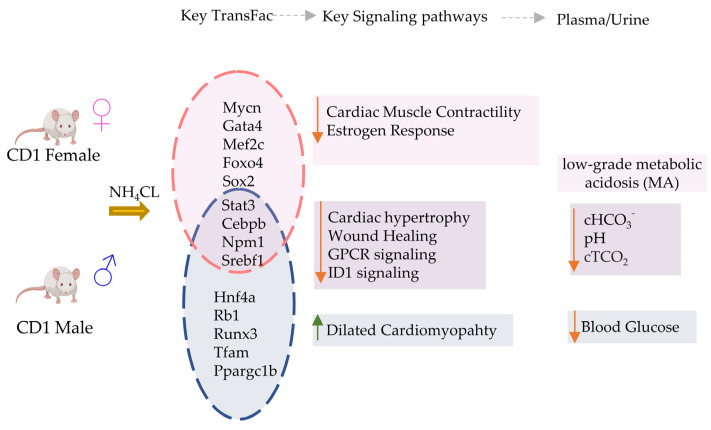
Summary of key transcriptional and electrolyte changes in male vs. female mice with acidosis. Differential regulation of transcriptional factors causes activation (Up arrow) or inhibition (down arrow) of key cardiac specific signaling pathways in male vs female mice. The blood chemistry was similar in both mice groups.

In this study, we performed a detailed transcriptomic analysis of cardiac tissue, in both male and female mice, to evaluate the effects of chronic low-grade MA on the heart (Figure 5). Although metabolic acidosis is observed in critically ill patients and results in several other chronic diseases, its direct effect on the heart, without associated co-morbidities such as heart failure or septicemia, is not fully understood. Our blood chemistry results indicate the successful induction of a low-grade MA in mice with NH_4_Cl administration, as published earlier [26], with a limited respiratory compensation, thereby providing an opportunity to investigate the direct effect of low-grade acidosis on the heart. The significant differences in gene expression in male and female mice, and a sexually dimorphic phenotype, are strongly indicative of a direct effect of low-grade acidosis on cardiac pathophysiology. This needs further validation through functional assessment of heart tissue at the organ and cellular levels.

Metabolic acidosis is characterized by systemic accumulation of acid, reduced bicarbonate (HCO_3_^−^) ions, and is a disorder that accompanies many primary clinical causes including diabetes, chronic kidney disease, surgery, cancer, a high protein diet, pharmacological drugs, and aging [86,87,88,89]. Disruption of the key buffering systems can result in high-grade acidosis. However, pH remaining balanced at values closer to the lower limit of the normal range (7.35) is categorized as low-grade acidosis. Although MA affects a very large proportion of the population, the effect of chronic MA, even low-grade, has not been well studied in many organs, particularly the organs with limited regenerative capacity that are more vulnerable to chronic changes in acid levels. The heart is one such organ which, apart from having very limited regenerative capacity, is also a metabolically highly active organ. Although indications are shown in the heart in response to acute or chronic MA, including arterial dilatation, hypotension, reduce cardiac output, and impaired immune response [90,91], little is understood about how MA transforms cardiac tissue, particularly at the transcriptomic levels that may inform changes in cardiac function. Furthermore, details about differences between the sexes in response to acidosis on cardiac tissue have not yet been described.

Clinical studies with chronic kidney disorder patients have shown an inconsistent association between reduced serum HCO_3_^−^ and cardiovascular events. While the AFDS (Australian Fremantle Diabetes Study) showed that reduced HCO_3_^−^ levels in serum had a significant association with increased risk of cardiovascular events [92], the Chronic Renal Insufficiency Cohort (CRIC) study did not show an increased heart failure rate to be associated with reduced serum HCO_3_^−^ levels [93]. A large retrospective study on non-dialysis chronic kidney disorder covering more than 50,000 patients showed that lower serum HCO_3_^−^ was associated with a higher risk of MACE+ (major adverse cardiovascular events) incidence [24]. However, the mechanisms by which MA causes cardiovascular impairment are unclear. We therefore sought to understand the transcriptomic changes taking place in the heart in response to MA, and to test whether changes in gene expression could inform cardiovascular disease manifestation.

Our MA model is a physiologically mimicking mouse model of acute low-grade acidosis, recreating the slight decrease in blood pH, without significant change in blood gas levels, but with a marked decrease in base excess of extracellular fluid, as well as a base excess of blood. Furthermore, in our previous work, we have shown that our model also mimics loss in body weight and bone loss, as well as loss in bone mineralization, that commonly accompanies acidosis [26].

There is an increased appreciation of the sexual dimorphism in cardiac disease manifestation [94]. Although females develop cardiovascular diseases 7 to 10 years later than males do, they are still the largest cause of death in females over 65 years of age [95]. It is likely that female hormones provide cardioprotection; however, the post-menopause age group has a high risk profile for cardiovascular diseases [96,97]. The pathophysiology of cardiovascular diseases also differs between males and females, with females more likely to present with heart failure accompanying preserved ejection fraction (HFpEF), a growing pathology with a high mortality burden and lack of therapies [98]. Women have distinct heart physiology, with a smaller left ventricular chamber and increased elastance in systolic and diastolic cycles, which predisposes them to HFpEF [99]. Owing to these and many other well-established sex differences in cardiac biology [100], we used both male and female mice to test the effect of low-grade acute (2 week induction) acidosis on the cardiac tissue.

Overall, our findings show that a small number of key genes change in expression in the heart in response to MA, but these changes can manifest differently in male and female hearts. Remarkably, for transcription factor activations, we found that most predicted TFs were either affected in males only, or in females only, in response to acidosis. This was a very surprising observation, because many of these TFs are key regulators of cardiovascular phenotypes. These TFs include GATA4, MEF2C, MYCN, FOXO4, RUNX1, RUNX3, MEF2C, CITED2, TFA4, HIF1A, SMAD2, CEBPB, NCOA1 and others, regulating a large number of downstream genes (Figure 5). How MA is regulating the downstream transcriptional outcome of these TFs in such a dimorphic way is certainly worthy of deeper exploration. Estrogen signaling, as well as other female hormones, are known to provide cardioprotection via multiple mechanisms [101]. Estrogen protects the female heart from ischemic, cytotoxic, and hypertrophic stimuli [102]. Estrogens are known to provide protection against acidosis-induced damage to chondrocytes [103]. In cancers, where the microenvironment can be highly acidic owing to high lactate production [104], estrogen receptor signaling is differentially regulated in females, providing protection against epithelial to mesenchymal signaling [105]. An interesting question therefore arises: are female hormones acting differently in a slightly acidic environment in MA, resulting in sexually dimorphic cardiovascular consequences?

Finally, we note that MA changes gene expression related to several cardiac pathologies differently in males and females, and sometimes in an opposing fashion. Of note was a clear activation of gene sets related to dilated cardiomyopathy, particularly in males, while response to estrogen was inactivated, particularly in females. We also found that the cardiac contractility related gene set was activated in males, while it was inactivated in females, in response to acidosis. We have carefully analyzed the most informative cardiac specific genes in these sets, showing that acidosis can significantly change the expression of these genes, likely contributing to disease phenotype.

Our study is a transcriptomic analysis of the cardiac tissue in response to systemic low-grade acidosis, which affects hundreds of millions of people worldwide. The study raises immediate questions about the mechanisms by which reduced pH affects cardiovascular gene expression in such a dramatic manner, and presents avenues for further research on the effects of acidosis on cardiac function, either in mice models or in mature human cardiac constructs that recreate an adult-like phenotype. Our study also highlights the large differences in cardiovascular disease manifestation between males and females in response to acidosis, suggesting sex-specific mechanisms at play in how low serum pH impairs cardiac function.

## Data Availability

Data will be available on request, and will be deposited to a public repository.
